# Diagnosing Polyparasitism in a High-Prevalence Setting in Beira, Mozambique: Detection of Intestinal Parasites in Fecal Samples by Microscopy and Real-Time PCR

**DOI:** 10.1371/journal.pntd.0005310

**Published:** 2017-01-23

**Authors:** Lynn Meurs, Anton M. Polderman, Natalie V. S. Vinkeles Melchers, Eric A. T. Brienen, Jaco J. Verweij, Bernhard Groosjohan, Felisberto Mendes, Manito Mechendura, Dagmar H. Hepp, Marijke C. C. Langenberg, Rosanne Edelenbosch, Katja Polman, Lisette van Lieshout

**Affiliations:** 1 Department of Parasitology, Leiden University Medical Center, Leiden, The Netherlands; 2 Department of Biomedical Sciences, Institute of Tropical Medicine, Antwerp, Belgium; 3 Faculty of Health Science, Catholic University of Mozambique, Beira, Mozambique; Centers for Disease Control and Prevention, UNITED STATES

## Abstract

**Background:**

Many different intestinal parasite species can co-occur in the same population. However, classic diagnostic tools can only frame a particular group of intestinal parasite species. Hence, one or two tests do not suffice to provide a complete picture of infecting parasite species in a given population. The present study investigated intestinal parasitic infections in Beira, Mozambique, i.e. in the informal settlement of Inhamudima. Diagnostic accuracy of five classical microscopy techniques and real-time PCR for the detection of a broad spectrum of parasites was compared.

**Methodology/Principal Findings:**

A cross-sectional population-based survey was performed. One stool sample per participant (n = 303) was examined by direct smear, formal-ether concentration (FEC), Kato smear, Baermann method, coproculture and real-time PCR. We found that virtually all people (96%) harbored at least one helminth, and that almost half (49%) harbored three helminths or more. Remarkably, *Strongyloides stercoralis* infections were widespread with a prevalence of 48%, and *Ancylostoma* spp. prevalence was higher than that of *Necator americanus* (25% *versus* 15%), the hookworm species that is often assumed to prevail in East-Africa. Among the microscopic techniques, FEC was able to detect the broadest spectrum of parasite species. However, FEC also missed a considerable number of infections, notably *S*. *stercoralis*, *Schistosoma mansoni* and *G*. *intestinalis*. PCR outperformed microscopy in terms of sensitivity and range of parasite species detected.

**Conclusions/Significance:**

We showed intestinal parasites—especially helminths—to be omnipresent in Inhamudima, Beira. However, it is a challenge to achieve high diagnostic sensitivity for all species. Classical techniques such as FEC are useful for the detection of some intestinal helminth species, but they lack sensitivity for other parasite species. PCR can detect intestinal parasites more accurately but is generally not feasible in resource-poor settings, at least not in peripheral labs. Hence, there is a need for a more field-friendly, sensitive approach for on-the-spot diagnosis of parasitic infections.

## Author Summary

In populations living in adverse conditions due to poverty, a wide variety of intestinal parasite infections can be observed. These infections are usually diagnosed by stool microscopy, but can be easily missed if the procedures used are inaccurate or performed in a suboptimal way. In the present study, we investigated the prevalence of intestinal parasite infections in an informal settlement in Beira, Mozambique. We also compared the diagnostic performance of five different microscopic techniques and real-time PCR for the detection of DNA of five helminth and five protozoa species. For this purpose, a single stool sample was collected from members of 63 households, evenly distributed within the settlement. Parasitic infections were found to be highly abundant in the 303 examined samples. Virtually all individuals were found to be infected with at least one helminth species and a majority of the stools showed multiple parasites. None of the classical microscopic techniques was suitable to detect all different pathogenic species. Hence, the outcome of several microscopy procedures had to be combined to get a complete picture. We found PCR to be the most accurate diagnostic approach, even in this hyper-endemic setting. However, DNA detection is a high-tech technology, generally not applicable in resource-poor settings.

## Introduction

Intestinal parasitic infections are among the most prevalent infections in humans in low- and middle-income countries. They can be largely categorized into two groups, i.e. helminthic and protozoan infections. Intestinal parasitic infections can cause significant morbidity. Especially children—who are generally more prone to heavy worm burdens—suffer from the sequelae of intestinal parasitic infections, such as diarrhea, malabsorption and anemia [1;2].

The most important intestinal helminths, both in terms of abundance and disease burden, are soil-transmitted helminths (STHs) such as hookworms, *Ascaris lumbricoides*, and *Trichuris trichiura* [3]. It is estimated that STHs infect more than two billion people or more than a third of the world’s population [4]. Also, the *Schistosoma* spp. blood flukes are of great public health importance, with more than 250 million people infected worldwide [5;6], and an estimated global disease burden of 4.0 million disability-adjusted life years (DALYs) [7].

In comparison to helminth infections, less is known about intestinal protozoan infections. They have been associated with persistent diarrhea in developing countries [8–10], and can cause severe morbidity, especially in immunocompromised individuals [11]. Hundreds of millions of people may be affected by intestinal protozoa annually [12;13]. Yet, there are no reliable estimates of the global burden of disease [14–16]. This lack of knowledge is due to the fact that intestinal protozoa are difficult to diagnose. Also, some diagnostic techniques cannot distinguish pathogenic from non-pathogenic species (i.e. *Entamoeba histolytica* versus the other *Entamoeba* spp.). For some species there is no consensus on their pathogenicity (e.g. *Blastocystis*), while for others, disease only develops in certain infected individuals but not in all (e.g. *Giardia intestinalis*). Loss of microscopic skills in many clinical laboratories and the general lack of awareness on protozoon infections further add to these difficulties.

The diagnosis of intestinal parasites typically relies on the microscopic detection of egg, larval, trophozoite, cyst, and/or oocyst life stages in human feces samples [17;18]. The sensitivity of stool microscopy is generally low, and for a reliable diagnosis it is important to choose the appropriate microscopic technique [19]. For example, relatively simple techniques such as the direct smear are known to detect high *A*. *lumbricoides* loads while underestimating the presence of other helminths such as *Schistosoma mansoni* [20]. Ideally, the technique with the highest diagnostic accuracy for the parasite of interest should be selected. In practice however, this is difficult to achieve since many different parasite species may occur in a given population, or even in a single individual, and resources are generally limited in countries where most of these infections are endemic, so not all appropriate microscopic techniques can be used. In the past decade, alternative diagnostic procedures have become available, such as the detection of parasite DNA in stool samples using real-time PCR [21]. The disadvantage of PCR, however, is that—in contrast to microscopy—it needs a high-tech laboratory, which is even more of a challenge for diagnostic laboratories within endemic countries.

Relatively little is known about the distribution of intestinal parasites in Mozambique [22;23]. The present study was initiated because a local hospital noticed many cases of diarrhea in one of the informal settlements (‘bairro’) in Beira, Mozambique. Given the sanitary conditions in the study area, intestinal parasites were suspected to be the cause of these complaints. However, diagnostic methods that were being used in the hospital at that time were not adequate to detect these infections. Hence, the aim of this study was 1) to investigate which intestinal parasite species are most common in this area, and 2) to compare diagnostic accuracy between different microscopic techniques and real-time PCR for these intestinal parasitic infections. Five commonly used microscopic techniques were applied and evaluated, i.e. direct smear, formal-ether concentration (FEC), Kato smear, Baermann method, and coproculture, for the detection of a uniquely broad spectrum of intestinal parasites: from intestinal helminths like *Strongyloides stercoralis*, *Ancylostoma* spp., *Necator americanus*, *A*. *lumbricoides*, *T*. *trichiura* and *Schistosoma* spp. blood flukes, to pathogenic intestinal protozoa such as *G*. *intestinalis*, *E*. *histolytica*, the coccidium *Cystoisospora belli* and the microsporidia *Enterocytozoon bieneusi* and *Encephalitozoon* spp. Microscopy and real-time PCR results were compared to one another and to composite reference standards (CRSs).

## Materials and Methods

### Ethics statement

Approval to perform the study was obtained from the Beira Committee of Medical Ethics, Mozambique and the study proposal was filed by the Committee of Medical Ethics of the Leiden University Medical Centre (reference number CI5.151/NV/ib). Prior to the study, written informed consent was obtained from the head of participating households. Individuals who were infected according to microscopy were offered treatment following standard clinical practice at the local hospital. Samples were anonymized for further data analysis.

### Study population

The study was performed in Inhamudima (E34.86°, S19.84°), an informal settlement in the city of Beira, Mozambique, and was conducted on request of the local hospital and faculty of medicine. The area of Inhamudima is frequently flooded and is not connected to a sewage system. The rainy season lasts from October to March. The study was performed between June and August 2007.

**Fig 1:**
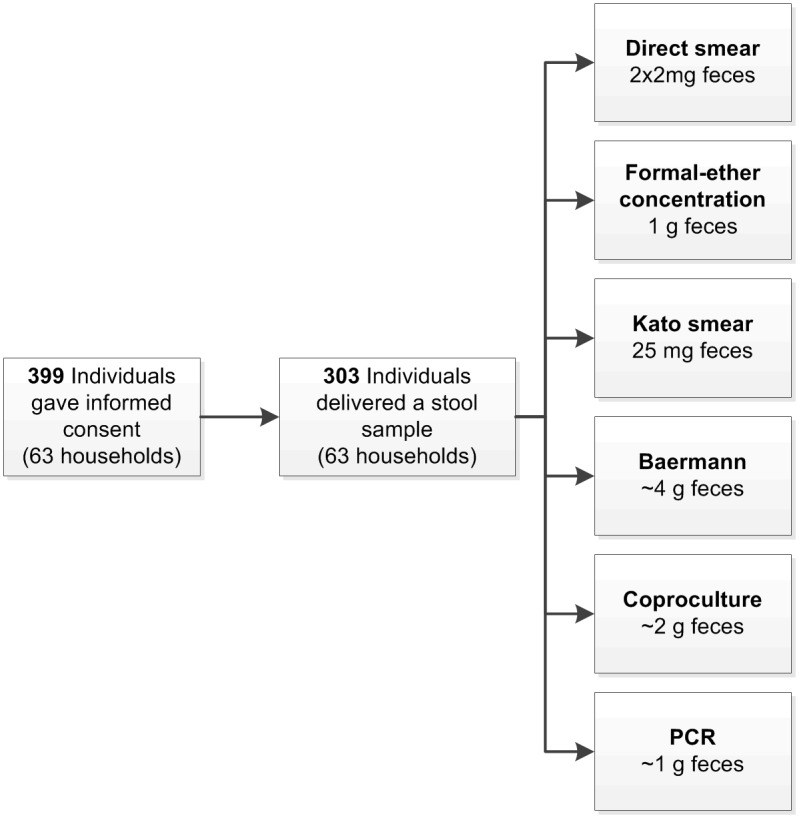
Flow diagram of the selection of the study population and diagnostic procedures.

A geographical map of this area was prepared and households and roads were annotated. In order to obtain a random and geographically evenly distributed sample of households and a logistically feasible sample size, a grid with 75 x 75 meter quadrants was superimposed on this map and the household that was closest to each of the intersections was selected. In this way, all participants of in total 63 households were approached to participate. In the field, these houses were located using handheld GPS devices. Infants (younger than one year) and people who did not provide sufficient fecal material for all procedures were excluded from the study. Fig 1 shows that 303 out of the 399 individuals that had given informed consent provided sufficient fecal material for inclusion into the study (i.e. participation rate of 76%).

Initially, urine samples were also collected for detection of *Schistosoma haematobium* (by urine filtration on one 10ml urine sample). Because of the relatively low numbers of *S*. *haematobium* cases however, collection of urine samples was stopped to focus on the diagnosis of intestinal parasites.

### Microscopy

Fecal samples were collected from all participating household members on a door-to-door basis, 0-18h after production of the samples, and examined in Beira within 24h after collection. Three well trained microscopists performed the laboratory procedures, and on average not more than eight stool samples were processed per day to ensure high quality microscopic results. Multiple approaches were used for the detection of cysts and oocysts of the protozoa, and eggs and larvae of the helminths (Table 1). Microscopic techniques included direct smear, FEC, Kato thick smear, Baermann method, and charcoal plate coproculture [17].

**Table 1:** Diagnostic methods performed and definitions of composite reference standards.

Parasite species		Direct smear	FEC	Kato smear	Baermann	Coproculture	PCR
STHs	*Strongyloides stercoralis*	+	+	-	+	+	+
	*Hookworm*	+	+	+	-	+	+ ^a^
	*Ascaris lumbricoides*	+	+	+	-	-	+
	*Trichuris trichiura*	+	+	+	-	-	-
*Schistosoma mansoni* ^b^		+	+	+ ^c^	-	-	+ ^d^
Pathogenic protozoa	*Giardia intestinalis*	+	+	-	-	-	+
	*Entamoeba histolytica* complex ^e^	+	+	-	-	-	+
	*Cryptosporidium parvum/C*. *hominis* ^f^	-	-	-	-	-	+ ^g^
	*Enterocytozoon bieneusi*	-	-	-	-	-	+ ^g^
	*Encephalitozoon* spp.	-	-	-	-	-	+ ^g^
	*Cystoisospora belli* ^f^	+	+	-	-	-	-
	*Cyclospora cayetanensis* ^f^	-	-	-	-	-	-
Non-pathogenic protozoa	*Entamoeba coli*	+	+	-	-	-	-
	*Entamoeba hartmanni*	+	+	-	-	-	-
	*Iodamoeba bütschlii*	+	+	-	-	-	-
	*Endolimax nana*	+	+	-	-	-	-
	*Chilomastix mesnili*	+	+	-	-	-	-
	*Blastocystis* ^h^	+	+	-	-	-	-
	*Dientamoeba fragilis* ^h^	-	-	-	-	-	-

FEC, formal-ether concentration

For each parasite species, the composite reference standard (CRS) is based on the combined findings of the test(s) indicated with a plus sign (+). The minus sign (-) indicates the tests that were not applicable for a given parasite species. The latter tests were not included in the respective CRSs.

^a^ In contrast to the microscopic techniques, the PCR differentiates between the hookworm species *Ancylostoma* spp. and *Necator americanus*.

^b^ In addition to the microscopic methods mentioned, glycerin sedimentation was carried out [17]. However, this was stopped after the first 100 samples because it was very labor-intensive and did not detect any additional *S*. *mansoni* cases.

^c^ The time interval between preparation of the Kato smear and the examination of the slides was 30–60 minutes and therefore suboptimal for the detection of *S*. *mansoni*.

^d^ The PCR detects DNA of both *S*. *mansoni* and *S*. *haematobium*.

^e^ *Entamoeba histolytica* cannot be differentiated microscopically from *E*. *dispar* and the other *Entamoeba* spp. of the *E*. *histolytica* complex: *E*. *bangladeshi*, *E*. *ecuadoriensis*, *E*. *moshkovskii*, and *E*. *nutalli* [24–26]. The PCR was specific for *E*. *histolytica*. Consequently, this parasite was not included in the analysis on diagnostic accuracy.

^f^ The microscopic methods used—direct smear and FEC—are known to be inadequate for these protozoan infections. Hence, the modified Ziehl-Neelsen staining [17] was performed but stopped after the first 201 samples because it was labor-intensive and *Cryptosporidium* was detected in three samples only, while *C*. *belli* and *C*. *cayetanensis* were absent in this subsample. Because of incompleteness the modified Ziehl-Neelsen staining, data was not included in the CRS.

^g^ These protozoa cannot be detected by any of the microscopic techniques used on all samples, and were therefore not included in the analysis on diagnostic accuracy.

^h^ As yet, the classification of *Blastocystis* and *D*. *fragilis* as pathogenic or non-pathogenic species remains controversial [27;28].

For the direct smear, ~2mg of feces was mixed with normal saline on a microscopy slide and examined for helminth eggs. Another ~2mg of feces was mixed with a drop of iodine and examined for protozoan cysts [17]. For FEC, the fecal parasite concentrator (FPC, Evergreen) was used. One gram of fecal material was thoroughly mixed with 8 ml of 10% formalin. An FPC strainer with 15 ml tube was attached to the tube containing this mixture. After having filtered the suspension into the empty tube, 3 ml of ether was added to the filtrate. This mixture was then shaken vigorously for 1 minute and centrifuged at 500 x *g* for 2 minutes. A thick, unstained wet mount of the sediment was used for the detection of helminth eggs and larvae. For protozoan cysts, a thin, iodine-stained wet mount of the sediment was used.

The Kato smear—also known as Kato-Katz smear—consisted of a single slide of fecal material [18;29;30]. A 25 mg template was placed on the microscopy slide and filled with sieved (~300 μm pore size) fecal material. Upon removal of the template, the sample was covered with a cellophane slip soaked with glycerol and water (1:1). The sample was flattened by pressing it onto an even surface, and examined 30–60 minutes after preparation.

For the Baermann method, fecal material (~4g) was placed on a layer of 2 hydrophilic gauze bandages. The gauze was folded into a pouch by attaching the four perforated corners of the gauze to a stick. Subsequently, the pouch was placed in a 50 ml tube filled with tap water for 3h in such a way that the pouch lightly touched the water. Most of the water was decanted and the remaining sediment was left to stand for 2 hours before being examined for nematode larvae.

For coproculture, the classical charcoal culture procedure was used [31]. Approximately 2g of fecal material was homogenized, mixed 1:1 with vermiculite, and placed on a filter paper on a plastic platform in a petri dish. Tap water was added to wet the filter paper and the petri dish was covered. After incubation at room temperature for 7 days, the water was collected in a tube and left standing for 2h. The sediment was examined for nematode larvae.

For the Baermann method as well as for coproculture, two microscopy slides were prepared, each with 100μl of the sediment. A drop of iodine was added if moving larvae were detected, enabling identification and quantification of the larvae.

### Real-time PCR

In Beira, an aliquot (~1g) of each stool sample was sieved and mixed with 3 volumes of 96% ethanol for preservation and shipment to Leiden, the Netherlands [32]. Here, the samples were stored at -20°C until detection and quantification of parasite DNA loads by real-time PCR. DNA isolation, amplification and detection were performed blinded to previous microscopic results.

For DNA isolation, 250μl of feces suspension was centrifuged and the pellet was washed with phosphate-buffered saline, resuspended in 200μl of 2% polyvinylpolypyrolidone (Sigma) and heated for 10 minutes at 100°C [32;33]. After sodiumdodecyl sulfate-proteinase K treatment (2h at 55°C), DNA was isolated using QIAamp Spin Columns/Mini Kit (Qiagen, Germany). In each sample, a fixed amount of Phocine Herpes Virus 1 was included within the isolation lysis buffer as an internal control [34].

In total, 10 PCR targets were included and 5 μl DNA was used in each real-time PCR. Amplification generally comprised of 15’ at 95°C followed by 50 cycles of 15” at 95°C, 30” at 60°C, and 30” at 72°C. Parasite-specific primers and probes were used for amplification of sequences, according to previously published protocols. Hookworm DNA (*Ancylostoma* spp. and *N*. *americanus*) was detected by one multiplex PCR described by Verweij et al. [35]. *Ascaris lumbricoides* [36] and *S*. *stercoralis* [37] DNA was detected in separate PCRs instead of in a multiplex format combined with other helminth targets. Schistosomal DNA was detected in an additional PCR as described by Obeng et al. [38–41]. Protozoa (*E*. *histolytica*, *G*. *intestinalis*, and *C*. *parvum/C*. *hominis*) DNA was detected by multiplex HGC-PCR [42]. Microsporidial (*E*. *bieneusi* and *Encephalitozoon* spp.) DNA was detected in another multiplex PCR described by Verweij et al. [43].

Negative and positive control samples were included in each PCR run. The PCR output from this system consisted of a cycle-threshold (Ct) value, representing the amplification cycle in which the level of fluorescent signal exceeded the background fluorescence. Hence, low Ct values correspond to high parasite-specific DNA loads in the sample tested, and vice versa. The maximum Ct value was set at 50 indicating that DNA was not detected in the sample after 50 cycles of amplification. The Ct values of the internal Phocine Herpes Virus 1 control were within the expected range (Ct value between 30 and 33) for all samples, indicating that there was no evidence of inhibition of amplification in any of these samples.

### Analysis

IBM SPSS 22.0 (IBM Corp.) and Microsoft Excel 14.0 (Microsoft Corp.) were used for statistical analyses. GraphPad Prism 5 (GraphPad Software, Inc.) was used to prepare graphs.

There is no gold standard for the detection of individual intestinal parasite species. Although microscopic techniques are known to lack sensitivity, they are, just as the PCR, supposed to be 100% specific [44]. We therefore combined the results of several diagnostic methods into a composite reference standard (CRS) [45;46]. The CRS was defined in such a way that it was negative if none of the diagnostic methods detected the parasite of interest, and positive if one or more methods detected the parasite. Table 1 shows how the CRS was defined for the different parasite species.

Infection prevalence was based on the CRS unless stated otherwise, and 95% Wald confidence intervals were calculated for this parameter. For sensitivities of the different diagnostic methods, the Wilson score method without continuity correction was used to calculate 95% confidence intervals [47]. Differences between test sensitivities were considered statistically significant if there was no overlap of their confidence intervals. The independent samples Mann-Whitney U test was used to determine whether differences in Ct values between microscopy-positives and -negatives were statistically significant.

## Results

### Characteristics of the study population

The study population (n = 303) consisted of 144 (48%) males and 159 (52%) females with a median age of 17 years (range 1 to 72). These people were derived from 63 households. Per household 1 to 11 subjects participated (median of 4 subjects).

### Infection prevalence

**Fig 2:**
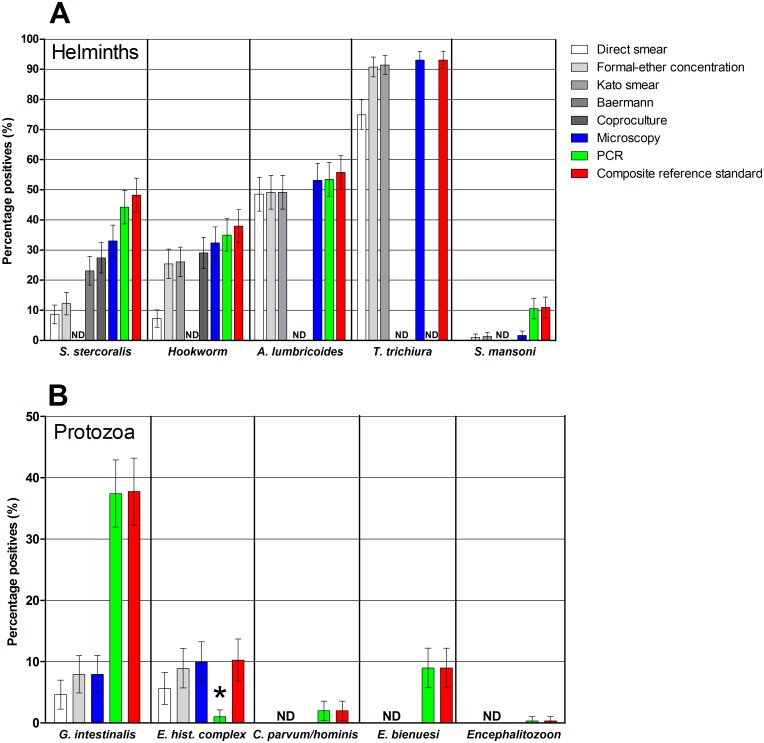
Prevalence of intestinal parasitic infections in the study population according to different diagnostic methods. Whiskers indicate 95% confidence intervals of the observed prevalence. Percentages are based on observations in 303 individuals. (A) Prevalence of helminth infections. *Strongyloides stercoralis* infection was not determined (ND) in Kato smears, hookworm was not determined by the Baermann method, while *A*. *lumbricoides*, *T*. *trichiura*, and *S*. *mansoni* were not determined by the Baermann method or coproculture. *Trichuris trichiura* was not determined by PCR either, and the composite reference standard (CRS) for this infection was consequently based on microscopic results only. (B) Prevalence of intestinal protozoan infections. Feces were examined by both microscopy and PCR for *G*. *intestinalis*, and *E*. *histolytica* complex spp. (one observation was missing for PCR, and consequently for the CRS). Only PCR data was used for *C*. *parvum/C*. *hominis* (one observation missing) and for, *E*. *bieneusi* and *Encephalitozoon* spp. (two observations missing).* While microscopy cannot differentiate between the pathogenic species *Entamoeba histolytica* and the nonpathogenic species of the *E*. *histolytica* complex, PCR is specific for the pathogenic species (*E*. *histolytica*).

STH infections were widespread with a prevalence of 93%, 56%, 48% and 38% for *T*. *trichiura*, *A*. *lumbricoides*, *S*. *stercoralis* and hookworm, respectively (Fig 2A). PCR indicated that *Ancylostoma* spp. was the most abundant hookworm: 25% of the population (75/303) harbored *Ancylostoma* spp. while *N*. *americanus* was detected in 15% (46/303) of the population. Mixed *Ancylostoma* spp. and *N*. *americanus* infections were observed in 5% (15/303) of the population. The prevalence of *S*. *mansoni* was 10.9%.

**Fig 3:**
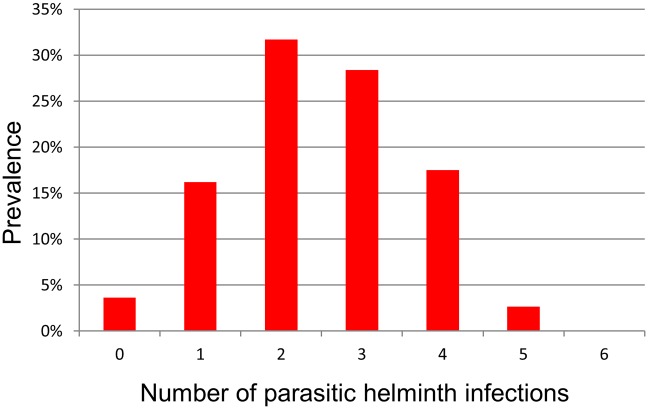
Number of helminth species found per person. Prevalence of infection is based on the composite reference standard for *S*. *stercoralis*, *A*. *lumbricoides*, *T*. *trichiura*, and *S*. *mansoni*, and on PCR for hookworm—*Ancylostoma* spp. and *N*. *americanus* (n = 303).

Within the population of Inhamudima, 96% (292/303) of individuals were found to harbor at least one of the following helminths in their stool sample: *S*. *stercoralis*, *Ancylostoma* spp., *N*. *americanus*, *A*. *lumbricoides*, *T*. *trichiura*, *S*. *mansoni*, and 49% (147/303) of the total population harbored three or more different helminth species (Fig 3). Up to five helminth infections were found in one individual.

The prevalence of infections with pathogenic protozoa is shown in Fig 2B. The pathogenic protozoan *Cystoisospora belli* was not detected in this population. *Giardia intestinalis* was the most common pathogenic protozoan with a prevalence of 37% (113/302). Microsporidia were found in 9% (28/301) and mainly consisted of *E*. *bieneusi* infections (27/28 cases). *Cryptosporidium parvum/C*. *hominis* was found by PCR in 2% (6/302) of the study population. All showed a Ct value higher than 30 (median Ct 37.6) and none of these 6 overlapped with the 3 samples positive in the Ziehl-Neelsen staining (legend f, Table 1). The prevalence of *E*. *histolytica* complex spp. infections was 10% (30/303) based on microscopy. However, PCR showed that only 10% (3/30) of these infections involved *E*. *histolytica*, i.e. the pathogenic species. Combining the findings of pathogenic protozoa with the detection of helminths, 98% (294/300) of the tested inhabitants of Inhamudima were found to be infected with at least one intestinal parasite species.

The prevalence of non-pathogenic protozoa varied between 4% and 34% for the different species, with a prevalence of 34% (102/303) for *Entamoeba coli*, 26% (80/303) for *Endolimax nana*, 21% (63/302) for *Blastocystis*, 9% (26/303) for *Entamoeba hartmanni*, 8% (24/303) for *Chilomastix mesnili* and a prevalence of 4% (13/303) for *Iodamoeba bütschlii*.

### Comparison of microscopic techniques

**Fig 4:**
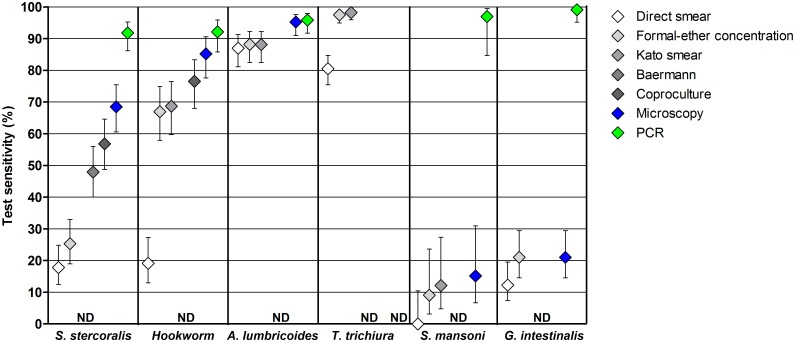
Sensitivities of the different diagnostic methods for the detection of intestinal parasitic infections. Whiskers indicate 95% confidence intervals of observed sensitivities (n = 303). *Strongyloides stercoralis* infection was not determined (ND) in Kato smears, hookworm was not determined by the Baermann method, while *A*. *lumbricoides*, *T*. *trichiura*, and *S*. *mansoni* were not determined by the Baermann method or coproculture. *Trichuris trichiura* was not determined by PCR, and for this species the sensitivity was therefore based on microscopic results only. *Giardia intestinalis* was not determined by Kato smear, the Baermann method or coproculture (one observation missing).

Diagnostic sensitivity was estimated for the different microscopic techniques and for each of the parasite species (Fig 4). FEC and Kato smear had the highest sensitivities for the detection of each of the helminths, except for *S*. *stercoralis*. The direct smear was inferior to these two methods for the detection of *S*. *stercoralis*, hookworm, *T*. *trichiura* and *S*. *mansoni*. Similarly, the direct smear was inferior to FEC for the detection of *G*. *intestinalis*. The Baermann method and coproculture showed the highest sensitivities for *S*. *stercoralis* (48% and 57%, respectively), while the direct smear and FEC showed equally low sensitivities (18% and 25%, respectively). The sensitivity of coproculture for the detection of *S*. *stercoralis* and hookworm (57% and 77%, respectively) tended to be higher than the sensitivity of the other microscopic techniques (up to 48% and 69%, respectively). Moreover, the sensitivity of helminth detection increased upon combination of multiple microscopic methods.

### Microscopy versus PCR

The sensitivity of PCR for the detection of each of the parasite species tested was higher than that of any of the microscopic techniques applied (Fig 4). This difference was statistically significant for all microscopic methods used for *S*. *stercoralis*, hookworm, *S*. *mansoni*, and *G*. *intestinalis*, and for the direct smear for the detection of *A*. *lumbricoides*. For some species, the sensitivity of the best microscopic technique was manifold lower than that of PCR. For example, the estimated sensitivity for the detection of *S*. *mansoni* was 12% for Kato smear versus 97% for the detection of *Schistosoma* DNA in feces via PCR. For *G*. *intestinalis*, sensitivity of FEC was 21% while that of PCR was 99%. In addition, in only one of the three PCR-positive *E*. *histolytica* samples, *E*. *histolytica* complex spp. cysts could be detected by microscopy (FEC).

**Fig 5:**
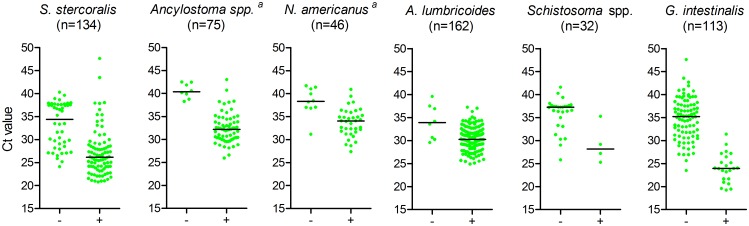
Ct values in PCR-positives: microscopy-negative *versus* -positive samples. Total number of PCR-positives per species is indicated between brackets. ‘-’ indicates microscopy-negative and ‘+’ microscopy-positive samples. Horizontal lines indicate median Ct values. Differences in Ct values between microscopy-positive and–negative samples were all significant p≤0.007, ^a^ microscopy cannot differentiate the two hookworm species.

Fig 5 shows that, for each parasite species, PCR-positive but microscopy-negative samples had significantly lower DNA loads (i.e. higher Ct values) than PCR-positive samples that were also microscopy-positive. Although observed less frequently, some microscopy-positive samples could not be confirmed by PCR. In most of these samples, only few parasites were detected by microscopy (see Table 2).

**Table 2:** Discordance between microscopy and PCR in microscopy-positives.

Parasite species	Number of microscopy-positive cases being PCR-negative (number of parasites observed per technique)						Total number of microscopy-positives
	Direct smear	FEC	Kato smear	Baermann	Coproculture	Total	
*S*. *stercoralis*	1 (1)	0	NA	7 ^a^	5 (6–141) ^a^	12	100
Hookworm	0	1 (2) ^b^	3 (1–47) ^b^	NA	7 (2–1000) ^b^	9	98
*A*. *lumbricoides*	2 (1)	5 (1–4)	0	NA	NA	7	161
*S*. *mansoni*	0	0	1 (1) ^c^	NA	NA	1	5
*G*. *intestinalis* ^d^	0	1 (1–10)	NA	NA	NA	1	24

FEC, Formal-ether concentration; NA, Not applicable

^a^ One case was detected by coproculture as well as the Baermann method. In addition to stool, Baermann medium and coproculture medium were analyzed by PCR in the 7 Baermann-positives and 3/5 coproculture-positives, respectively. These samples also tested negative.

^b^ One case was detected by FEC, Kato smear as well as coproculture.

^c^ In addition, 3 *S*. *haematobium* eggs were observed

^d^ Parasite numbers were determined semi-quantitatively.

## Discussion

The present study was initiated because a local hospital noticed many cases of diarrhea in Inhamudima. Given the adverse living conditions in the study area, intestinal parasites were suspected to be the cause of these complaints. However, diagnostic methods that were being used in the hospital at that time were not adequate. Clinical records thus far suggested that there were only low numbers of intestinal parasite infections in the area. The first aim of the present study was to obtain a comprehensive overview of the intestinal helminth and protozoan infections that occur in the informal settlement of Inhamudima in Beira.

The major strength of the present study is the unprecedentedly large panel of different diagnostic techniques used [48]. This diagnostic rigor resulted in high quality data regarding a uniquely wide spectrum of intestinal parasite species, including helminths as well as protozoa. Prevalences observed here are thus likely to approach the true prevalence of individual parasitic infections as well as co-infections [49], in contrast to other prevalence studies where generally only one microscopic technique is used. This approach led to several important observations concerning the presence of intestinal parasite infections in the target population, such as the remarkably high levels of *S*. *stercoralis*, and high parasite infection levels in general, as well as a high degree of polyparasitism.

According to Schär et al., only 44% of African countries have prevalence data on *S*. *stercoralis*, and a majority of these studies is based on inadequate diagnostic techniques [50]. In Mozambique, so far, only Mandomando et al. reported on *S*. *stercoralis*. A prevalence of 1.1% was reported in under-five-year-olds with diarrhea referred to a district hospital in Southern Mozambique, but only direct smear was used which is ineffective for the detection of this parasite [51]. As a result, *S*. *stercoralis* prevalences were assumed to be low, i.e. 6.2% prevalence in Mozambique [50]. Our results (prevalence of 48%) indicate that this may be a gross underestimation of the nationwide prevalence and once more illustrate how often strongyloidiasis is overlooked due to poor diagnostics.

Another important observation was that *Ancylostoma* spp. was more prevalent than *N*. *americanus* (25% *versus* 15%, respectively). This is in line with the studies described by Goldsmid [52], as well as a more recent study in pre-school children living in a rural area in Malawi where *Ancylostoma* spp. prevailed over *N*. *americanus* [53]. Despite of these reports, it is often assumed that only *N*. *americanus* is endemic in East Africa [54].

High levels of intestinal parasitic infections as observed in the present study are not exceptional in urban informal settlements. More than 20 years ago, it was already predicted that ongoing urbanization in the developing world would increase levels of intestinal parasites in areas like Inhamudima [55]. In such adverse living conditions, widespread environmental contamination is likely to occur for parasites such as *A*. *lumbricoides*, *T*. *trichiura*, and *G*. *intestinalis* [55]. The present observations confirm this idea as these three parasites ranked in the top four of most prevalent parasites in Inhamudima, with a prevalence as high as 93% for *T*. *trichiura*. Augusto et al. (2009) also noted a particularly high STH prevalence in (peri-)urban areas of Mozambique (including Beira, although these were not explicitly reported), and these prevalences were higher than those reported in older studies [22]. The authors hypothesized that the increase of STH infections in Mozambican informal settlements was due to rural-urban migration, low levels of socioeconomic development, and overcrowding together with the deterioration of water supply and sewage systems after independence [22].

In addition to determining infection levels of intestinal parasites in the inhabitants of Inhamudima, this study aimed to compare diagnostic accuracy between five microscopic techniques that are commonly used for the detection of intestinal parasites. The common microscopic techniques direct smear, FEC, Kato smear, Baermann method, and coproculture were applied and compared for the detection of parasitic helminths as well as protozoan infections. The classical broad-spectrum techniques FEC and Kato smear proved useful for the detection of intestinal helminths: hookworm, *T*. *trichiura* and *A*. *lumbricoides*. The Kato smear may be more informative than FEC as it is a quantitative technique, and in accordance with a recent meta-analysis by Nikolay et al., it tended to detect more STH infections—more hookworm, *T*. *trichiura*, and *A*. *lumbricoides*—than FEC [44]. On the other hand, the Kato smear is not suitable for the detection of pathogenic intestinal protozoa. In this respect, the two techniques are complementary. In order to also detect *S*. *stercoralis*, coproculture and/or the Baermann method should be added to the diagnostic work-up. Consistent with a recent meta-analysis by Campo Polanco et al. [56], coproculture tended to have a higher sensitivity for the detection of *S*. *stercoralis* than the Baermann method. We demonstrated that coproculture has the additional advantage that it has a higher sensitivity for the detection of hookworm than the other techniques, while the Baermann method is suited for the detection of *S*. *stercoralis* only. This was also reported by Knopp et al. [48]. The disadvantage of coproculture however, is that it takes a week to obtain test results while the Baermann method takes several hours, and that it is more difficult to perform under field conditions [48]. In addition, coproculture harbors the occupational hazard of infection by L3 larvae. In concordance with earlier reports [44], the direct smear was inferior to all the other diagnostic tests, for each of the endemic intestinal parasite species. This confirms that the direct smear is not at all suitable for the diagnosis of intestinal parasite infections. In practice however, it is widely used in endemic areas, in both research and clinical settings—including the Beira hospital—resulting in gross underestimations of the spread of intestinal parasites.

We aimed to compare the diagnostic accuracy of the different microscopic techniques to that of real-time PCR. The detection of parasite-specific DNA had a higher sensitivity than microscopy for *S*. *stercoralis*, hookworm, *Schistosoma* spp., and *G*. *intestinalis*, even when results from the five microscopic techniques were combined. These findings are in line with earlier studies, despite differences in endemicity and geographic locations [44]. For example, Arndt et al. showed the same helminth PCRs that were used in the present study (in a multiplex format [36]) to be more sensitive for the detection of *S*. *stercoralis*, hookworm and *Schistosoma* spp. than the combination of direct smear, FEC, and Kato smear [57]. Also Easton et al. recently reported that real-time PCR outperformed Kato-Katz for the detection of soil-transmitted helminths and has the advantage of demonstrating parasite species which are not diagnosed by Kato-Katz such as *G*. *intestinalis*, *E*. *histolytica* and *S*. *stercoralis* [58]. Earlier findings from our group comparing Kato smear and PCR in Kenyan schoolchildren showed that PCR outperformed the Kato smear for the detection of *N*. *americanus* as well as *S*. *mansoni*. Even when three consecutive stool samples were analyzed using Kato smear, while PCR was performed on only one stool sample, PCR detected more cases [41;59].

When focusing on the diagnosis of *Schistosoma* only, previous studies found PCR to be more sensitive than microscopy, [41], also when using other *Schistosoma*-specific targets [60–64]. It should be noted that *S*. *haematobium* was detected in our study populations. This parasite was seen in six out of 95 examined urine samples (6%). PCR results indicated that *Schistosoma* DNA was absent in stool samples from people with *S*. *haematobium* eggs in urine but without *S*. *mansoni* infection according to microscopy. In accordance with the Kenyan study [41], *S*. *haematobium* co-endemicity is thus unlikely to explain the relatively low sensitivity of microscopy as compared to PCR for the detection of *S*. *mansoni*. Yet, we cannot entirely rule out that “leakage” of *S*. *haematobium* DNA may have resulted in occasional false-positive PCRs.

To our knowledge, only one study reported a lower diagnostic accuracy of PCR relative to microscopy. Knopp et al. showed real-time PCR for the detection of hookworm and of *S*. *stercoralis* to be less sensitive than microscopy when applied on stool samples collected in a low-endemicity area in Tanzania [65]. Although this study used the same PCR procedures as in the present study, this inconsistency could still be explained by minor technical differences in the performance of the DNA extraction or the general PCR set-up, resulting in a reduced test efficiency which becomes more obvious when infection levels are already low. Hence, standardization of laboratory procedures and the implementation of external quality assessment schemes are warranted.

It is widely recognized that PCR could be particularly useful for the detection of intestinal parasites in low transmission areas and in post-control settings [48;58]. Here, we show that PCR can also have added value in high transmission areas, particularly in polyparasitized populations. It not only has a higher sensitivity than microscopy for all the intestinal parasites investigated in this study, but it also has the advantage that it can differentiate between morphologically identical species where microscopy cannot. This is important because infections caused by species passing morphologically indistinguishable eggs, such as the hookworms, can have different epidemiology. Moreover, it also detects infections such as microsporidia which are extremely difficult to diagnose by conventional light microscopy. Although it was not yet possible to detect *T*. *trichiura* by PCR at the time of study, currently available multiplex PCRs do include this helminth. PCR may thus be a useful tool for evaluation of public health interventions, for quality control of microscopy procedures as well as for research on the distribution of intestinal parasitic infections in different endemic settings [46;57]. However, it should be noted that PCR also has disadvantages. PCR consumables are expensive, and it requires high-tech equipment which is often not available in laboratories in affected regions. Transfer of samples to other, better equipped laboratories, may prolong the turnaround time of PCR results. Secondly, the clinical relevance of submicroscopic intestinal parasitic infections is not yet fully understood, and therefore remains a matter of debate [16;57;66]. It has also been proposed that residual DNA may persist and be detected by PCR after parasite clearance [67], while others have showed fast DNA clearance after treatment [68;69]. More research is needed to clarify these issues.

### Conclusion

We demonstrate that intestinal helminth and protozoan infections and co-infections are widespread in Inhamudima, Beira. We showed that classical techniques such as FEC are useful for the detection of some intestinal helminths such as hookworm, *T*. *trichiura* and *A*. *lumbricoides*. However, they lack the sensitivity to reliably characterize the wide range of intestinal parasites that may coexist in a population or individual. PCR can detect intestinal parasites more accurately but in most endemic areas it is not (yet) possible to perform this technique, at least not in the more peripheral laboratories. So, until a more field-friendly approach becomes available, infection levels of intestinal parasites—and polyparasitism—are best approximated by combining multiple and relatively simple microscopic techniques.

## Supporting Information

S1 Checklist (DOCX)

S1 Database (XLSX)
